# Smartphone‐based Ecological Momentary Assessment to study “scanxiety” among Adolescent and Young Adult survivors of childhood cancer: A feasibility study

**DOI:** 10.1002/pon.5935

**Published:** 2022-04-25

**Authors:** Lauren C. Heathcote, Sarah J. Cunningham, Sarah N. Webster, Vivek Tanna, Elia Mattke, Nele Loecher, Sheri L. Spunt, Pamela Simon, Gary Dahl, Marta Walentynowicz, Elizabeth Murnane, Perri R. Tutelman, Lidia Schapira, Laura E. Simons, Claudia Mueller

**Affiliations:** ^1^ Health Psychology Section, Department of Psychology, Institute of Psychiatry, Psychology, and Neuroscience, King's College London London UK; ^2^ Department of Anesthesiology, Perioperative, and Pain Medicine, Stanford University School of Medicine Stanford California USA; ^3^ Department of Mental Health Law and Policy University of South Florida Tampa Florida USA; ^4^ Stanford Cancer Institute, Stanford University School of Medicine Stanford California USA; ^5^ Lucile Packard Children's Hospital at Stanford Palo Alto California USA; ^6^ Centre for the Psychology of Learning and Experimental Psychopathology, KU Leuven Leuven Belgium; ^7^ Psychological Science Research Institute Université Catholique de Louvain Louvain‐la‐Neuve Belgium; ^8^ School of Engineering Dartmouth College Hanover New Hampshire USA; ^9^ IWK Health Center Halifax Nova Scotia Canada; ^10^ Department of Pediatrics, Stanford University School of Medicine Stanford California USA

**Keywords:** cancer, childhood cancer survivors, Ecological Momentary Assessment, oncology, psycho‐oncology, scanxiety

## Abstract

**Objective:**

Scan‐related anxiety (“scanxiety”) refers to the fear, stress, and anxiety in anticipation of tests and scans in follow‐up cancer care. This study assessed the feasibility of Ecological Momentary Assessment (EMA) for real‐world, real‐time capture of scanxiety using patients' personal smartphone.

**Methods:**

Adolescent and Young Adult survivors of childhood cancer were prompted to complete EMA surveys on a smartphone app three times per day for 11 days (33 surveys total) around their routine surveillance scans. Participants provided structured feedback on the EMA protocol.

**Results:**

Thirty out of 46 contacted survivors (65%) enrolled, exceeding the preregistered feasibility cutoff of 55%. The survey completion rate (83%) greatly exceeded the preregistered feasibility cutoff of 65%. Participants generally found the smartphone app easy and enjoyable to use and reported low levels of distress from answering surveys. Participants reported significantly more daily fear of cancer recurrence (FCR) and negative affect in the days before compared to the days after surveillance scans, aligning with the expected trajectory of scanxiety. Participants who reported greater FCR and scanxiety using comprehensive measures at baseline also reported significantly more daily FCR around their surveillance scans, indicating validity of EMA items. Bodily threat monitoring was prospectively and concurrently associated with daily FCR, thus warranting further investigation as a risk factor for scanxiety.

**Conclusions:**

Findings indicate the feasibility, acceptability, and validity of EMA as a research tool to capture the dynamics and potential risk factors for scanxiety.

## 1 BACKGROUND

Medical scans and tests occur repeatedly throughout active cancer treatment and can continue for years after treatment ends. Surveillance scans assess for disease progression, treatment response, cancer recurrence and treatment sequalae. For survivors of childhood cancer, there is prolonged and sometimes lifelong assessment for late effects of treatment. The term “scanxiety” was first coined in 2011 to reflect the fear, stress, and anxiety that accompanies scans and awaiting news of their results.^1^ Although scanxiety can be considered a normal reaction to a repeated stressor, for many it is a considerably negative experience that impairs quality of life^2^, ^3^ and thus warrants research and clinical attention.

Ecological Momentary Assessment (EMA) is an intensive longitudinal research method that permits real‐world, real‐time capture of patient experiences using their personal smartphone. Ecological Momentary Assessment is well‐suited to capture transitory and short‐lived but uniquely stressful periods in oncology care, such as surveillance testing and experiences of scanxiety. With EMA, patients can be prompted to complete brief surveys on their recent thoughts, affect, symptoms, and behaviors within semi‐randomized time windows throughout the day. Ecological Momentary Assessment could also be used to assess emotional reactivity around surveillance scans, thus capturing this component of scanxiety as it unfolds in real‐time. Ultimately, supportive oncology care could be made more effective with dynamic assessment of patients' experiences to help refine psycho‐oncology theory and guide the development of timely and targeted interventions.^4^, ^5^

Most studies of scanxiety have used cross‐sectional and retrospective methods to identify risk factors that are time‐invariant (e.g., lower education or a history of smoking) and confer risk for scanxiety over months or years.^3^ Instead, EMA allows for dynamic capture of scanxiety across the anticipatory phase (the days or weeks leading up to the scan) and the recovery phase (the days or weeks following the scan) to identify factors that confer risk or protection for scanxiety over shorter periods of time. Vigilance for and misinterpretation of bodily symptoms may confer short‐term risk for escalations in cancer‐related fears by triggering concerns of recurrence that are maintained via an anxious state of bodily monitoring and self‐checking behaviors.^6^, ^7^ Indeed, recent studies in Adolescent and Young Adult (AYA) survivors have shown that bodily symptoms such as pain can trigger fear of cancer recurrence (FCR) years after finishing treatment.^8^, ^9^, ^10^ Ecological Momentary Assessment could be effectively used to capture this bodily threat monitoring and its dynamic interactions with FCR over time. While EMA has been demonstrated as a feasible and acceptable method in both cancer populations and in youth, it has been less studied within the context of stressful waiting periods within clinical care. Oncology surveillance visits pose a unique challenge for capturing EMA data for several reasons, centrally that patients may be unwilling to complete such an intensive study design during a period of high medical uncertainty. Moreover, it could be a challenge to keep participants engaged during the days after receiving good news, which is a period that can be characterized by high re‐engagement with life and a parallel reprieve from and disengaging with the topic of cancer.

Thus, the goal of this study is to assess the feasibility, acceptability, and validity of EMA as a research tool to study scanxiety among AYA survivors of childhood cancer. Regarding the EMA protocol, we operationalize scanxiety as Negative Affect (NA), stress, and FCR around surveillance scans. First, we assessed the feasibility of recruitment, and participant engagement, for this time‐intensive study design. Second, we assessed the validity of the EMA surveys to capture scanxiety. Specifically, we examined whether participants reported greater daily FCR, stress, and NA in the days before as compared to the days after surveillance scans; this would align with the predicted pattern of scanxiety. We also examined whether participants who reported greater FCR and scanxiety using comprehensive baseline measures would also report greater daily FCR, stress, and NA across the study period; this would speak to the validity of the EMA items. Finally, we explored bodily threat monitoring as a potential risk factor for scanxiety. We preregistered a priori feasibility metrics, and primary and secondary study aims on Researchgate.com prior to the start of data collection.^11^

## 2 METHODS

### 2.1 Recruitment

This study was approved by the Institutional Review Board at Stanford University School of Medicine (IRB‐55183). Participants were recruited from the Bass Center for Childhood Cancer and Blood Diseases from March to July 2021. Eligible participants were identified by screening medical records or referral from an oncology clinician.

### 2.2 Participants

Adolescent and Young Adult survivors were eligible to participate if they (1) were 11–25 years old, (2) had received a previous cancer diagnosis (3) had completed active cancer treatment of curative intent, (4) were proficient in English, and (5) owned or had access to a smartphone. For survivors under 18 years, caregivers provided consent and survivors provided assent. Survivors 18 years and older provided consent. Given the pilot feasibility nature of the study, we aimed to recruit a sample of *N* = 30.

### 2.3 Procedure

Study enrollment and training were completed remotely via Zoom.^12^ Upon enrollment, participants completed an online baseline survey using REDCap, a secure on‐line data acquisition system.^13^ Baseline surveys were completed an average of 5 days before the EMA protocol. Six days before their scheduled follow‐up appointment, participants downloaded an EMA app, LifeData,^14^ to their smartphone. Participants were prompted to complete three surveys per day, for 5 days before, on the day of, and for 5 days after their oncologist appointment in which they received results of their surveillance scans (11 days, 33 surveys total). A time‐contingent sampling design was used, in which surveys were administered on a stratified schedule at a randomized time within three windows (morning survey: 8–10 AM, afternoon survey: 1–4 PM, evening survey: 6–9 PM). Up to three reminder notifications were sent, 20 min apart, until the survey was completed. Surveys closed 2 hours after the first notification, thus ensuring that surveys were not completed retroactively. Participants were compensated with $20 for completing the baseline questionnaires, $2.50 for each completed EMA survey, and a $25 bonus for completing all EMA surveys. Participants were informed that they could discontinue study procedures at any time, for example, if procedures caused them distress or if they experienced a cancer recurrence (of note, no participants experienced a cancer recurrence during this study).

### 2.4 Measures

#### 2.4.1 Baseline surveys

Participants completed baseline questionnaires reporting sociodemographics and psychosocial functioning; measures pertaining to the goals of this study are described below. Oncology history (diagnosis, treatment intensity) was gathered from patient medical records.

##### Fear of cancer recurrence (FCR)

At baseline, participants' FCR was assessed via the FCR Inventory – Child Version (FCRI‐C).^15^ The FCRI‐C was adapted from the short‐form version of the adult FCR Inventory (FCRI‐SF),^16^ using language that is more appropriate for youth aged 8 and older. The FCRI‐C comprises nine items, one of which is reverse scored. Scores range from 0 to 36, with higher scores indicating greater FCR.

##### Scanxiety

In this study, we have operationalized scanxiety as emotional reactivity and FCR captured in real‐time around surveillance scans. While there is no gold‐standard assessment of scanxiety, at baseline we also administered a static self‐report measure to capture the more cognitive components of scanxiety and to align with a previous study in adults.^2^ We adapted the Children's Revised Impact of Events Scale (CRIES‐8),^17^ an abbreviated child version of the Impact of Events Scale – Revised (IES‐R),^18^ to capture scanxiety in youth. The IES‐R was previously adapted by Bauml and colleagues^2^ to assess scanxiety in adults with lung cancer. The CRIES‐8 was anchored to surveillance scans; the instructions read “Below is a list of difficulties people sometimes have around stressful life events. Please indicate how frequently each statement was true for you in the run up to your most recent scan or cancer check‐up.” Scores range from 0 to 24, with higher scores indicating more severe scanxiety.

##### Bodily threat monitoring

The Bodily Threat Monitoring Scale (BTMS) is a new 19‐item self‐report measure that captures the tendency to monitor and interpret bodily sensations as symptomatic of something being wrong with one's body. Items include “*I monitor my body for signs that something is wrong”* (threat monitoring) and “*When I have a bodily sensation I can’t explain, I think it means that something is wrong with my body*” (threat interpretations). Scores range from 0 to 76, with higher scores indicating greater bodily threat monitoring. The BTMS showed excellent internal consistency (Cronbach's alpha = 0.95), acceptable test‐retest reliability (*r* = 0.77, *p* < 0.001), and evidence of construct validity (association with the Body Vigilance Scale; *r* = 0.60, *p* < 0.001) in a separate sample of childhood cancer survivors aged 10–25 years (manuscript in prep).

#### 2.4.2 EMA surveys

Ecological Momentary Assessment items are presented in full in Appendix A. We conducted cognitive interviews with two AYA survivors to adapt the language and content of the EMA items. All EMA items were phrased to capture participants' experiences since the previous survey.

**Fear of cancer recurrence** was assessed using two items adapted from the FCRI‐SF that captured the number of times that participants thought about cancer that day and the level of worry about cancer that day.^7^, ^19^ The FCR items were administered at the Evening survey only to alleviate emotional burden and reduce possible reactivity effects.

**Stress** was assessed three times per day using items from the Perceived Stress Scale adapted for momentary use.^20^

**Negative and Positive Affect (PA)** were assessed three times per day using five PA and 5 NA items adapted from the Positive And Negative Affect.^21^ We sought to retain adequate construct coverage of the original PANAS to include items that capture fearful/anxious, sad/depressed, and angry/hostile mood, as well as some type of PA.^22^ We chose not to use the item “energetic” as this was too overlapping with the symptom of fatigue and thus could artificially conflate affect and symptom scores.

**Bodily threat monitoring** was assessed three times per day by adapting two items from the BTMS for daily use. We adapted one item that captured monitoring of bodily symptoms and one item that captured worry about bodily symptoms.

**Self‐checking behaviors** were assessed during the Evening survey only. Participants were asked whether they physically examined themselves for signs of cancer that day (yes/no).

**Somatic symptoms** were assessed three times per day via 14 items that were collated across several clinical symptom measures to capture symptom severity across a range of cancer‐related and everyday symptoms.

**Social connectedness** was assessed three times per day via two items that captured the number of social interactions^23^ and perceived level of social support.^24^

##### Objective physical activity

Participants uploaded a screenshot of their ambulatory step count displayed in the Health App (iOS) or Google Fit App (Android) at the Evening survey only.

#### 2.4.3 Feedback survey

Participants reported on the acceptability of study procedures via 10 closed‐ended and three open‐ended questions which solicited additional feedback and suggestions for improving the study design (see Appendix B for the full feedback survey).

### 2.5 Data analysis

Our preregistered primary aim was to examine EMA feasibility in terms of recruitment and engagement, and acceptability of EMA study procedures in terms of participant feedback. Recruitment feasibility was determined via an a priori benchmark of 55%, and engagement feasibility was determined via an a priori benchmark of 65%; these benchmarks were selected based on a previous EMA feasibility study of cancer caregivers.^4^ Our preregistered secondary aims were to assess EMA item variance across time and the extent to which comprehensive questionnaires administered at baseline were prospectively associated with EMA responses; these were assessed via *t*‐tests and Pearson correlations. Ecological Momentary Assessment data was aggregated across the full 11 days as well as the first 6 days and the last 5 days, and these mean values were used as person‐level variables.

## 3 RESULTS

### 3.1 Sample characteristics and baseline scanxiety

As seen in Table 1, participants underwent a range of scans and medical tests during their surveillance visits, including X‐ray, CT, MRI, echocardiogram, and laboratory tests (e.g., complete blood count). At baseline, the mean (SD) CRIES‐8 score was 10.5 (5.65), with a range of 0–18. All but one participant (96.3%) showed some degree of scanxiety (i.e., scores >0) at baseline. Girls reported more severe scanxiety at baseline than boys (*t*(25) = 2.21, *p* = 0.04). All baseline associations between demographic, medical, and self‐report data are presented in Table S2 (see Appendix C).

**TABLE 1:** Sample characteristics

	*N* (%)	Mean
Sex		
Male	15 (50)	
Female	14 (47)	
Non‐binary	1 (3)	
Age (years)		17.6
11–14	5 (17)	
15–18	15 (50)	
19–25	10 (33)	
Ethnicity		
Hispanic, Latino/a, or Spanish origin	8 (26)	
Race		
White	20 (67)	
Asian	3 (10)	
Mixed race	6 (20)	
Other	1 (3)	
Diagnosis		
ALL/AML	9 (30)	
Solid tumor	7 (23)	
Lymphoma	13 (43)	
CNS tumor	1 (3)	
Medical history		
Age at diagnosis (years)		13.3
Time since treatment (months)		44.8
History of relapse (yes)	3 (10)	
Treatment intensity		
Least intensive	1 (3)	
Moderately intensive	13 (43)	
Very intensive	12 (40)	
Most intensive	4 (13)	

Of note, two patients had their oncology appointments rescheduled to a later date; both participants restarted the surveys 5 days before their updated appointment. For most patients their results were provided on the same day as the scan, but in some cases, additional scans and tests were conducted either the day before or the day after the oncology appointment.

### 3.2 Primary aim: Feasibility of EMA procedures

#### 3.2.1 Recruitment

**FIGURE 1:**
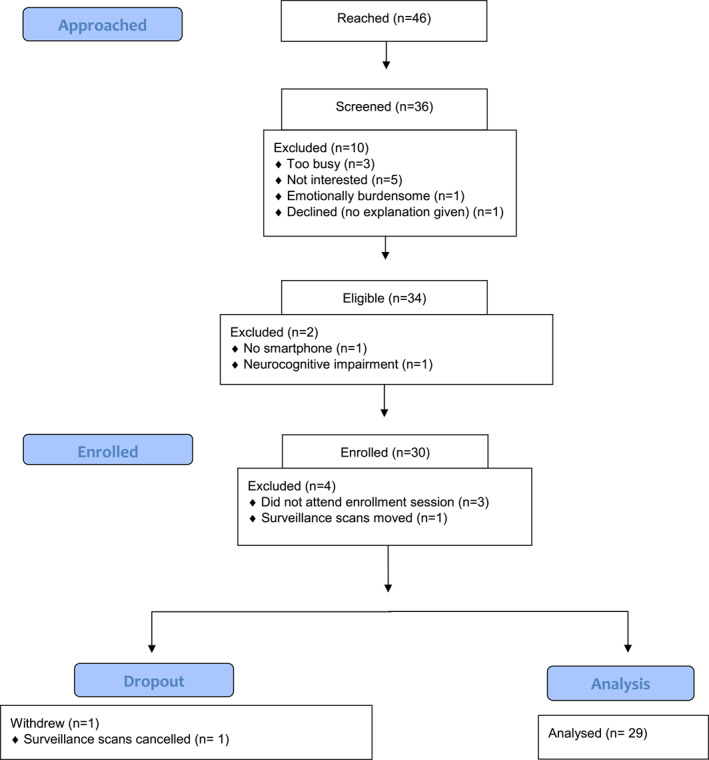
CONSORT diagram

We established contact with 46 AYA survivors to describe study procedures, of whom 10 (22%) declined screening. Of the 36 screened, two (6%) were ineligible (Figure 1). Of the 34 interested and eligible, four (12%) were not enrolled. The 30 enrolled participants represent 88% of confirmed eligible individuals and 65% of all individuals reached for recruitment, exceeding our a priori benchmark of 55% recruitment. One participant (3%) withdrew part way through the study due to their surveillance scans being cancelled as the family were relocating; when excluding this participant, the a priori benchmark for recruitment was still met (63%).

#### 3.2.2 Engagement

Of 957 survey prompts issued to 29 study completers, participants completed 789 surveys (overall completion rate = 83%; daytime surveys = 82%, nighttime surveys = 83%), exceeding the a priori benchmark of 65%. The median percentage of surveys completed by participants was 91%. Twenty‐four participants (83% of study completers) completed ≥65% of the surveys. The range of surveys completed was 42%–100%. Participants took an average (*M*) of 2 min and 33 s (*SD* = 1 min and 9 s) to complete each survey and received an average (*M*) of 1.10 reminders per survey (*SD* = 0.55; *Mode* = 0). On average, participants uploaded step count screenshots on 8 out of the 11 study days (range 0–11 days). Participants who reported significantly more FCR (baseline and EMA: *r* = 0.39, *p* = 0.04) and bodily threat monitoring (baseline: *r* = 0.56, *p* = 0.003; EMA: *r* = 0.43, *p* = 0.02) completed significantly more surveys. Survey completion rate did not differ by baseline levels of scanxiety (*r* = 0.28, *p* = 0.15), age (*r* = 0.13, *p* = 50), and gender (*t*(25) = 0.47, *p* = 0.64).

#### 3.2.3 Participant feedback

**FIGURE 2:**
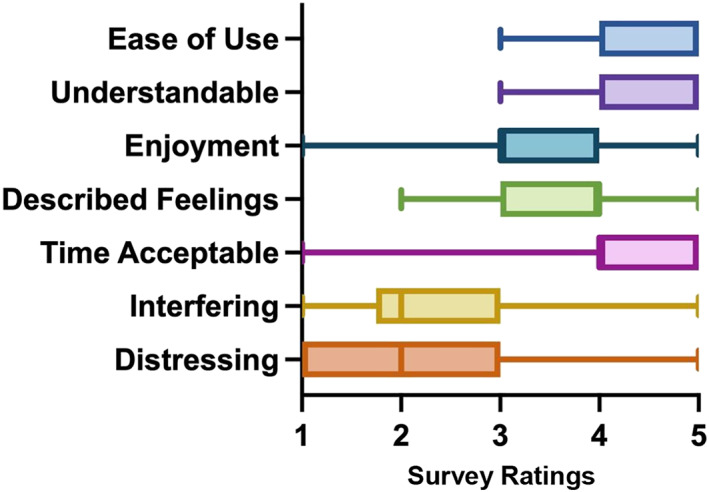
Acceptability of Ecological Momentary Assessment (EMA) protocol via quantitative feedback survey (see Appendix B for rating labels)

Of the 29 participants who completed the study, 26 (90%) completed the final feedback survey. As seen in Figure 2, quantitative feedback was generally positive. All 26 participants found the app easy or very easy to use, 25 found the questions easy or very easy to understand, and 21 found the amount of time it took to complete surveys was acceptable or very acceptable. Over two‐thirds (*N* = 18) found the questions helpful or very helpful in describing their daily feelings. Almost half (*N* = 12) found the app enjoyable or very enjoyable to use. Two‐thirds of participants reported that the app did not interfere or interfered very little in their daily activities; one participant reported that the app greatly interfered with their daily activities. Almost one quarter (*N* = 6) reported that it would have been acceptable to complete up to five surveys per day; almost half (*N* = 12) reported that this would be acceptable only if the surveys were shorter. Average levels of distress from completing surveys were low, and over half of participants (*N* = 14) reported very little or no distress. Three participants reported that they found it very distressing to answer survey questions.

Qualitative data generally reflected the quantitative data, with participants reporting that their overall experience was “*easy,*” “*pretty good,*” “*a great overall experience,”* and “*covered any type of feeling I would have had.*” Participants also indicated additional positive reactions to the study, for example that “*this was a really cool study*” and that it was “*extremely important*” and “*pretty cool to be apart [sic] of something.*” Some participants reported positive sentiments around the daily surveying of their experiences, for example that it “*helped me have a time during my day to focus on how I feel. It was nice to stop and think.*” Regarding more negative experiences, one participant described that they “*were mostly frustrated*”; this participant also reported that the surveys caused them distress.

### 3.3 Secondary aims: Validity of EMA surveys to capture scanxiety

#### 3.3.1 Do EMA responses follow the expected trajectory of scanxiety?

As seen in Table 2 and aligned with expectations, participants reported significantly greater FCR, NA, and bodily threat monitoring in the 6 days before surveillance scans compared to the 5 days after having received scan results. Unexpectedly, participants also reported significantly greater PA in the 6 days before compared to the 5 days after surveillance scans. Also unexpectedly, there was no significant difference in stress before compared to after surveillance scans.

**TABLE 2:** Mean Ecological Momentary Assessment (EMA) responses before and after surveillance scans

	Before scans		After scans			
	*M*	*SD*	*M*	*SD*	*t*	*p*
FCR	0.82	0.68	0.48	0.61	4.49	<0.001
Negative affect	0.66	0.63	0.51	0.57	2.31	0.011
Stress	1.65	0.81	1.72	0.82	−1.01	0.321
Bodily threat monitoring	0.69	0.69	0.50	0.56	3.45	0.002
Positive affect	1.91	0.83	1.69	0.95	2.73	0.011

#### 3.3.2 Do comprehensive baseline assessments predict EMA responses?

**FIGURE 3:**
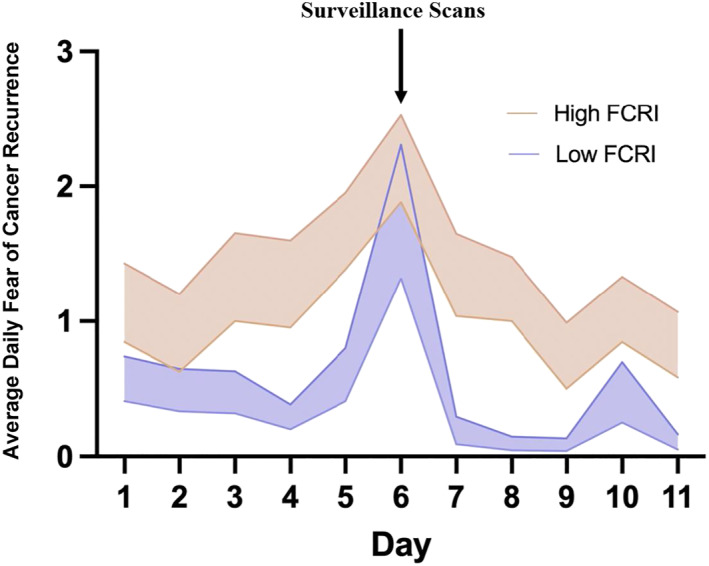
Daily fear of cancer recurrence (FCR) ratings for those with high versus low baseline Fear of Cancer Recurrence Inventory – Child Version (FCRI‐C) scores (*Mdn split* = 15.5)

As seen in Table 3, participants who reported greater FCR using the FCRI‐C also reported more average daily FCR and less average daily PA. Participants who reported greater scanxiety using the CRIES‐8 reported more average daily FCR and stress compared to those who reported less scanxiety at baseline. To further illustrate the association between baseline and daily FCR, Figure 3 shows the trajectory of daily FCR for those with low (i.e., below the median) versus high (i.e., above the median) scores on the FCRI‐C. Visual inspection of Figure 3 reveals that those with low FCR at baseline show a similar FCR peak on the day of surveillance scans as those with high FCR, but that those with high FCR at baseline show elevated FCR on the other days. Additionally, those with low FCR at baseline show a more rapid decline in FCR following surveillance results compared to those with high FCR at baseline.

**TABLE 3:** Associations between medical characteristics, baseline assessments, and averaged Ecological Momentary Assessment (EMA) responses

EMA									
		FCR	Negative affect	Stress	Bodily threat monitoring	Self‐checking behaviors	Positive affect	Social interaction	Social connection
Baseline	Age	0.175	**0.368^†^**	−0.071	−0.057	0.033	−0.031	**−0.361^†^**	**0.309^†^**
	Treatment intensity	−0.14	−0.200	0.196	−0.077	−0.006	−0.211	**−0.346^†^**	−0.092
	Time since Tx	0.191	0.012	−0.293	−0.004	0.140	0.410*	0.224	0.144
	FCR	0.734***	0.146	**0.355^†^**	**0.361^†^**	**0.327^†^**	−0.379*	**−0.373^†^**	−0.205
	Scanxiety	0.522**	0.164	0.435*	0.146	0.252	−0.291	−0.150	−0.111
	Bodily threat monitoring	0.568**	0.213	0.113	0.463*	0.443*	−0.098	−0.138	0.139

*Note*: Medium (*r* ≥ 0.30) and large (*r* ≥ 0.50) effects in bold.

***p < 0.001, **p < 0.01, *p < 0.05, and ^†^ *p* < 0.10.

#### 3.3.3 Is bodily threat monitoring concurrently and/or prospectively associated with scanxiety?

As seen in Table 3, participants who reported greater bodily threat monitoring at baseline using the BTMS reported more average daily FCR, less average daily PA, and engaged in more daily self‐checking behaviors. Within EMA responses, participants who reported more daily bodily threat monitoring also reported more daily FCR and NA, but not stress or PA.

## 4 DISCUSSION

We used an intensive smartphone‐based longitudinal research method (EMA) to capture experiences of scanxiety in AYA survivors of childhood cancer. The preregistered feasibility metrics of 55% enrollment and 65% survey engagement were surpassed, and feedback on the EMA protocol was generally positive. Participants' cancer‐related and general distress was higher in the days before compared to the days after surveillance scans, aligning with the predicted pattern of scanxiety and thus indicating validity of the EMA surveys. This finding also aligns with a previous study of women who had completed breast cancer treatment, in which FCR was found to increase before and decrease after the mammogram.^25^ Average EMA responses across the study period generally aligned with comprehensive assessments of FCR and scanxiety as assessed at baseline, further supporting validity of the EMA items. Overall, findings demonstrate the feasibility, acceptability, and preliminary validity of an 11‐day EMA protocol to study scanxiety.

On average, participants reported low levels of distress from answering survey questions. However, three participants (10% of the sample) reported high levels of distress. No participants withdrew from the study despite being informed that they could do so at any time if study procedures caused them distress. One reason for this may be that the compensation schedule was tied to survey completion rate. Additionally, participants may have chosen to tolerate distress for the perceived benefits of contributing to research that will help others, or to improve introspective understanding. Further research is needed to identify which EMA items cause distress, to identify participants who are most likely to experience distress, and to develop effective methods for supporting self‐efficacy in withdrawing from EMA studies.

Intensive longitudinal data from EMA designs enable the identification of factors that confer short‐term risk for elevations in scanxiety. We found that survivors who were generally more vigilant for and worried about bodily symptoms experienced more daily FCR, less PA, and performed more cancer self‐checking behaviors. These findings align with a previous study which found that FCR predicted more self‐checking behaviors in breast cancer survivors anticipating an upcoming mammogram.^7^ Taken together, heightened bodily threat monitoring, possibly accompanied by excessive self‐checking behaviors, warrants further investigation as a possible risk factor for real‐time elevations in scanxiety.

Our findings also offer insight into the prevalence of scanxiety. Aligning with a previous study using a similar baseline measure in adults with lung cancer,^2^ we found that scanxiety as assessed with the CRIES‐8 was common (96.3% reported some degree of scanxiety) and that females reported significantly more severe scanxiety than males. Unsurprisingly, we also found a significant association between baseline assessments of FCR (the FCRI‐SF) and scanxiety (the CRIES‐8). It is unclear, however, how scanxiety relates to FCR as a construct, the latter of which has been much more widely studied; this points to an important direction for future research.

### 4.1 Study limitations

First, there may have been sampling bias in that only individuals who were willing to tolerate repeated surveys during this stressful period would have enrolled. Second, as baseline FCR and scanxiety were assessed just prior to the EMA protocol, associations with EMA responses may have been conflated. Administering additional baseline questionnaires at a different time would overcome this issue. Third, FCR items did not explicitly ask survivors the extent to which they worried about a recurrence. We also did not include items from the CRIES‐8 within the EMA protocol. Instead, we operationalized more general affect and stress within the surveillance period as a way to capture scanxiety that is complimentary to these more targeted assessments. The acceptability of such direct FCR and scanxiety assessment in EMA studies remains to be demonstrated. Fourth, the preregistered recruitment benchmark of 55% was selected based on a previous study of cancer caregivers^4^ and thus does not directly align with the current population. Yet of note, our recruitment rate also surpassed that reported by Perndorfer and colleagues (29%) in their study of couples awaiting surveillance mammograms.^19^

### 4.2 Future research and clinical implications

There are several lines of investigation that could be pursued using this protocol. We know almost nothing about how quickly individuals recover after receiving reassuring scan results. Our findings indicate that FCR does not dissipate following reassuring scan results. Latency to distress reduction following a clear result may indicate need for supportive care. Additionally, our protocol assesses social connectedness which could be examined as a protective factor for scanxiety. Parental influences are particularly relevant for survivors of childhood cancer.^26^, ^27^ Alongside the objective step count assessments, use of wearables that capture heartrate and other smart devices that capture health behaviors (e.g., electronic bottle caps that monitor medication use^28^) could capture interactions of scanxiety with physiology and behavior. Mixed effects models with repeated measures, particularly modelling time as a quadratic function, will be particularly useful to probe these questions. Finally, EMA facilitates Ecological Momentary Intervention. Dynamic, in vivo assessment of scanxiety could facilitate the delivery of personalized and timely interventions, for example, by delivering coping strategies that target the survivors' symptom‐related concerns at the precise time that they occur.^5^

## 5 CONCLUSIONS

Findings indicate the feasibility and acceptability of EMA as a research tool, and the validity of the specific EMA surveys, to study scanxiety in AYA cancer survivors. Ecological Momentary Assessment could be effectively used to capture the dynamic antecedents and consequences of scanxiety, thus pointing towards risk and protective factors that could inform novel intervention targets.

## CONFLICT OF INTEREST

Lauren C. Heathcote has received consulting fees from Blue Note Therapeutics; this consultancy does not pertain to the current study. There are no other conflicts of interest.

## Supporting information

Supplementary Material S1

Supplementary Material S2

Supplementary Material S3

## Data Availability

The data that support the findings of this study are available from the corresponding author upon reasonable request.
