# Supplementary material for: Smartphone‐based Ecological Momentary Assessment to study “scanxiety” among Adolescent and Young Adult survivors of childhood cancer: A feasibility study
Source: Psychooncology. 2022 Apr 25;31(8):1322–30. doi: 10.1002/pon.5935 (PMC9545782; doi:10.1002/pon.5935)
Supplement: Supplementary file 1 — Supplementary Material S1 [file PON-31-1322-s001.pdf]

Appendix A
EMA Survey Items

Fear of Cancer Recurrence (2 items – EoD survey only)
→ How many times today did you think about cancer?
→ Response options:
- 0, No times
- 1, One time
- 2, Two times
- 3, Three times
- 4, Four or more times

→ How much did you worry about cancer today?
→ Response options:
- 0, Not at all
- 1, A little bit
- 2, Somewhat
- 3, Quite a bit
- 4, A whole lot

Cancer Self-Checking Behavior (1 item; EoD survey only)
→ Today, did you examine yourself physically for signs or symptoms of cancer?
→ Response options:
- 0, No
- 1, Yes

Negative Affect (5 items)
→ Since the last survey, to what extent have you felt:
- Irritable
- Mad
- Nervous
- Scared
- Sad

→ Response options for each:
- 0, Not at all
- 1, A little bit
- 2, Somewhat
- 3, Quite a bit
- 4, A whole lot

Positive Affect (5 items)
→ Since the last survey, to what extent have you felt:
- Excited
- Joyful
- Happy
- Proud
- Strong

→ Response options for each:

- 0, Not at all
- 1, A little bit
- 2, Somewhat
- 3, Quite a bit
- 4, A whole lot

Stress (3 items)

→Since the last survey, to what extent have you felt:

- Stressed
- That you can cope with things *(reverse scored)*
- That you're on top of things *(reverse scored)*

→ Response options for each:

- 0, Not at all
- 1, A little bit
- 2, Somewhat
- 3, Quite a bit
- 4, A whole lot

Bodily Threat Monitoring (2 items)

→ Since the last survey, to what extent did you do the following:

- I monitored my body for signs that something is wrong
- I worried about my bodily sensations

→ Response options for each:

- 0, Not at all
- 1, A little bit
- 2, Somewhat
- 3, Quite a bit
- 4, A whole lot

Somatic Sensations (14 items)

→ Since the last survey, to what extent have you felt:

- A headache
- A stomachache
- Chest tightness
- Sore throat or cough
- A runny or congested nose
- Faint or dizzy
- Shortness of breath
- Stiff or sore muscles
- Tired
- Nauseous or like you could vomit
- Numbness or tingling
- Itchy skin
- Heart pounding or racing
- Another bodily sensation or symptom (please write below)

→ Response options for each:

- 0-10 scale (“Not at all” to “Worst possible”)

Social Connectedness (2 items)

→ Since the last survey, how many times did you interact with someone else [e.g., spent more than five minutes talking/communicating with someone else]?”

→ Response options:

- 0, No interactions
- 1, 1 interaction
- 2, 2 interactions
- 3, 3 interactions
- 4, 4 or more interactions

→ Since the last survey, to what extent have you felt connected to other people?

→ Response options:

- 0, Not at all
- 1, A little bit
- 2, Somewhat
- 3, Quite a bit
- 4, A whole lot
