# Supplementary material for: Smartphone‐based Ecological Momentary Assessment to study “scanxiety” among Adolescent and Young Adult survivors of childhood cancer: A feasibility study
Source: Psychooncology. 2022 Apr 25;31(8):1322–30. doi: 10.1002/pon.5935 (PMC9545782; doi:10.1002/pon.5935)
Supplement: Supplementary file 2 — Supplementary Material S2 [file PON-31-1322-s002.pdf]

Appendix B
Feedback Survey

Close-Ended Questions:

a. How easy was the app for you to use?
    i. 1 – very difficult
    ii. 2
    iii. 3
    iv. 4
    v. 5 – very easy
b. How much did you enjoy using the app?
    i. 1 – not at all
    ii. 2
    iii. 3
    iv. 4
    v. 5 – very much
c. How understandable were the questions?
    i. 1. – difficult to understand
    ii. 2
    iii. 3
    iv. 4
    v. 5 – easy to understand
d. How helpful were the questions in describing your daily feelings?
    i. 1 – very unhelpful
    ii. 2
    iii. 3
    iv. 4
    v. 5 – very helpful
e. How distressing did you find it to answer the questions?
    i. 1 – not at all distressing
    ii. 2
    iii. 3
    iv. 4
    v. 5 – very distressing
f. Was the amount of time it took to complete the questions acceptable?
    i. 1 – very unacceptable
    ii. 2
    iii. 3
    iv. 4
    v. 5 – very acceptable
g. Would it have been acceptable to complete 5 surveys per day?
    i. Yes, this would be acceptable
    ii. Yes, but only if the surveys were shorter
    iii. No
h. How much did completing the questions interfere with your daily activities?
    i. 1 – interfered greatly

    ii. 2
    iii. 3
    iv. 4
    v. 5 – did not interfere

i. On average, how many times each day did you think about cancer throughout this study?
    i. No times
    ii. One time
    iii. Two times
    iv. Three times
    v. Four or more times

j. On average, how much did you worry about cancer each day, throughout this study?
    i. 0, Not at all
    ii. 1, A little bit
    iii. 2, Somewhat
    iv. 3, Quite a bit
    v. 4, A whole lot

Open-Ended Questions:

k. What was your overall experience of participating in this study?
l. When did you find it easiest and hardest to answer the questions?
m. Did you have any concerns arise while answering the questions?
n. What suggestions do you have for how we could make the experience better for future study participants?
