# Supplementary material for: Smartphone‐based Ecological Momentary Assessment to study “scanxiety” among Adolescent and Young Adult survivors of childhood cancer: A feasibility study
Source: Psychooncology. 2022 Apr 25;31(8):1322–30. doi: 10.1002/pon.5935 (PMC9545782; doi:10.1002/pon.5935)
Supplement: Supplementary file 3 — Supplementary Material S3 [file PON-31-1322-s003.docx]

**Appendix C**

**Additional Tables**

*Table S1.* Descriptive Statistics and Psychometric Properties of Focal Variables

|  | **Mean** | **SD** | **Range** | **Skew** | **α** | **ICC** | **# Items** |
| --- | --- | --- | --- | --- | --- | --- | --- |
| **Baseline variables** |  |  |  |  |  |  |  |
| Scanxiety (CRIES-8) | 15.25 | 9.19 | 0-28 | -0.07 | .81 | .34 | 8 |
| Fear of Cancer Recurrence (FCRI-C) | 15.25 | 6.22 | 5-26 | -0.04 | .80 | .30 | 9 |
| Bodily Threat Monitoring (BTMS) | 26.15 | 14.16 | 1-53 | 0.18 | .93 | .41 | 19 |
| **EMA variables** |  |  |  |  |  |  |  |
| Fear of Cancer Recurrence | 0.68 | 0.89 | 0-4.0 | 1.33 | .53 | .36 | 2 |
| Negative Affect | 0.60 | 0.76 | 0-3.8 | 1.79 | .76 | .39 | 5 |
| Stress | 1.66 | 0.94 | 0-2.8 | 0.09 | .56 | .30 | 3 |
| Bodily Threat Monitoring | 0.67 | 0.80 | 0-3.5 | 1.32 | .59 | .42 | 2 |
| Positive Affect | 1.78 | 1.01 | 0-4.0 | 0.50 | .78 | .41 | 5 |

*α = Cronbach’s alpha (*note:* α is person-centered for EMA variables to account for within-person nested data structure)

*Table S2.* Associations Between Baseline Demographic, Medical, and Self-Report Variables

|  | Treatment Intensity | Time Since Tx | FCR | Scanxiety | Bodily Threat Monitoring |
| --- | --- | --- | --- | --- | --- |
| Age | .27 | -.04 | .32 | .00 | .24 |
| Treatment Intensity |  | .10 | -.20 | -.34 | -.32 |
| Time Since Tx |  |  | -.12 | -.24 | .07 |
| FCR |  |  |  | .58** | .62*** |
| Scanxiety |  |  |  |  | .58** |

***p<.001, **p<.01
